# E-based physical exercise in patients with multiple sclerosis and comorbidity (COMPACT): feasibility study and protocol for a randomized controlled trial

**DOI:** 10.3389/fimmu.2026.1625017

**Published:** 2026-04-13

**Authors:** Rafl Adnan, Sara Samadzadeh, Camilla Richter, Sören Möller, Stine Gundtoft Roikjaer, Anne Froelich, Søren T. Skou, Ulrik Dalgas, Nasrin Asgari

**Affiliations:** 1 Institute of Regional Health Research, University of Southern Denmark, Odense, Denmark; 2 Department of Neurology, Slagelse Hospitals, Slagelse, Denmark; 3 Charité – Universitätsmedizin Berlin, corporate member of Freie Universität Berlin and Humboldt-Universität zu Berlin, Experimental and Clinical Research Center, Berlin, Germany; 4 The Research and Implementation Unit PROgrez, Department of Physiotherapy and Occupational Therapy, Næstved-Slagelse-Ringsted Hospitals, Slagelse, Denmark; 5 Epidemiology, Biostatistics and Biodemography, Department of Public Health, University of Southern Denmark, Odense, Denmark; 6 Innovation and Research Centre for Multimorbidity, Slagelse Hospital, Region Sjælland, Denmark; 7 Section of General Practice, Faculty of Health and Medical Sciences, University of Copenhagen, Copenhagen, Denmark; 8 Center for Muscle and Joint Health, Department of Sports Science and Clinical Biomechanics, University of Southern Denmark, Odense, Denmark; 9 Exercise Biology, Dep. Public Health, Aarhus University, Department of Public Health, Aarhus, Denmark; 10 Institute of Molecular Medicine, University of Southern Denmark, Odense, Denmark

**Keywords:** comorbidity, feasibility study, multiple sclerosis, physical exercise, randomized controlled trial

## Abstract

**Background:**

Comorbidity is prevalent among people with multiple sclerosis (pwMS) and may contribute to disease progression. Physical exercise (PE) reduces symptoms in pwMS and also benefits comorbidities. Digital (e)-based PE has been proposed as a tool to support the integration of PE.

**Aims:**

To describe a protocol for a randomized controlled trial (RCT) based on a structured approach and the results from a controlled feasibility study of an e-based PE intervention in pwMS with and without comorbidities.

**Method:**

In a RCT following a feasibility study (n=50), patients will be randomly assigned in a 1:1 ratio to receive either usual care (n=150) or usual care plus an e-based PE program at home (n=150). The exercise program consists of resistance training with resistance bands targeting the lower extremities. The sessions will enable participants to engage in group exercises from their homes, supervised online by physiotherapists, two 60-minute sessions per week for 6 months (24 weeks and 48 sessions). The primary endpoint is change of walking capacity using the 6-minutes’ walk test. Secondary endpoints include “no evidence of disease activity” (NEDA)-3 scale, measures of quality of life and fatigue as well as levels of neurofilament light chain in blood and cerebrospinal fluid.

Results of the feasibility study: Fifty individuals were eligible and randomized to an intervention group (n = 23) or to a usual care control group (n = 27). A total of 24 sessions were conducted for three months in groups of 6, supervised by the physiotherapist. In the intervention group, two pwMS did not begin the PE program due to occupational constraints with a resulting recruitment rate of 91.3% (21/23). Of the remaining 21 individuals, 16 (76.2%) completed the follow-up assessments with a mean attendance of 15.2 (range 6-22) sessions per participant, corresponding to a 63% adherence rate. No intervention‐related adverse events were reported.

**Conclusion:**

This protocol describes a prospective RCT study and the supporting feasibility data of an e-based PE performed at home. The effects of e-based PE performed at home will be evaluated, offering a significant contribution to the field of digital healthcare solutions and MS.

**Clinical Trial Registration:**

https://clinicaltrials.gov/, identifier NCT06298201.

## 1 Introduction

Multiple sclerosis (MS) is a potentially disabling inflammatory demyelinating disease of the central nervous system (CNS). It manifests as a consequence of immune attacks on myelin and nerve fibers which leads to neuronal damage (neurodegeneration) and subsequently to the clinical accumulation of neurological disability (1–5).

Over the last two decades, the age-specific prevalence of people with MS (pwMS) has increased substantially (6–8), and aging will expectedly amplify the risk of comorbidities and conversion into a progressive disease course (9). Comorbidity refers to the total burden of any additional entity other than the specific index disease (10, 11) Physical inactivity is a major risk factor for the development of comorbidity in pwMS (10). Emerging evidence underscores the high prevalence of comorbidity in pwMS, evident even at the time of MS diagnosis (10, 12). This is of importance as comorbidities can lead to delayed MS diagnosis, accelerated disease progression, and diminished efficacy and safety of MS treatments, subsequently increasing mortality (10, 13–15). Cardiovascular disorders comorbidities and related risk factors (RF) including type 2 diabetes mellitus (T2DM), obesity, hyperlipidemia and hypertension are prevalent among pwMS (12, 16).

Physical exercise (PE) is advocated as an effective treatment for at least 26 chronic diseases, including MS (17–19). In pwMS, PE positively modulate peripheral inflammatory markers such as TNF-alpha, INF-gamma, IL-4, IL-10, etc. (20–22), while also reducing the relapse rate (23). PE improves muscle strength and cardiorespiratory fitness (24–26), as well as walking speed, endurance (27–30), and balance in MS patients (31). Furthermore, PE can induce reductions in fatigue (32–40) and symptoms of depression, and improvements in cognitive dysfunction as well as quality of life in MS patients (35, 41–47). In pwMS, walking impairments are highly prevalent and up to 68% of the patients experience some degree of ambulatory dysfunction (48). Since walking is a cornerstone in the ability to perform activities of daily living, and to retain working capacity (49), walking impairment also impact quality of life (50). The 6-Minute Walk Test (6MWT) is considered the gold standard (51), and able to predict habitual walking in pwMS (52).

Despite the benefits of PE for MS patients, a significant proportion drop out or fail to maintain an active lifestyle post-rehabilitation (53, 54). This trend is a serious challenge for integrating and sustaining PE in the daily lives of pwMS. Consequently, solutions that can support pwMS in sustaining a physically active lifestyle are warranted. A promising category of potential solutions covers eHealth devices and digital healthcare tools (55–59). The effectiveness of e-based MS rehabilitation, including telerehabilitation, has shown promising results in improving fitness, gait, balance, and upper limb function, often equaling or exceeding traditional treatment (60–64). Excluding pwMS from clinical trials based on the presence of comorbidity is common, which consequently leads to limited reports about the effect of comorbidities on outcomes of rehabilitation trials (65). Further research is required to investigate e-based PE’s effectiveness and long-term impact on managing pwMS with comorbidities. Significant knowledge gaps exist on the effect of PE on pwMS and comorbidities. The adaptation of exercise to comorbidity evidently adds challenges and complexity as to the application and integration of behavior change techniques, which may be applied to the exercise plan. Furthermore, most existing studies have excluded pwMS that had comorbidities at baseline (66). Accordingly, guidance and guidelines are not yet available for pwMS with comorbidity.

The aim of this randomized clinical trial (RCT) is to investigate if e-based PE, in addition to usual care, will be more effective than usual care alone in improving health outcomes for pwMS with and without comorbidities. Because the intervention focuses on progressive resistance training of the lower extremities, the 6-Minute Walk Test (6MWT) was chosen as the primary endpoint, reflecting expected improvements in walking endurance and functional mobility. Secondary endpoints capture disease activity and broader health impact, including NEDA-3 (relapses, EDSS progression, MRI activity), as well as measures of cardiorespiratory fitness (Ekblom–Bak test), accelerometer-measured activity, quality of life, fatigue, mood, sleep, pain, and biomarkers such as levels of neurofilament light chain (NfL).We hypothesize that PE significantly reduces disability, increasing walking capacity as the primary outcome for pwMS and comorbidity.

## 2 Methods

### 2.1 Study design

**Figure 1:**
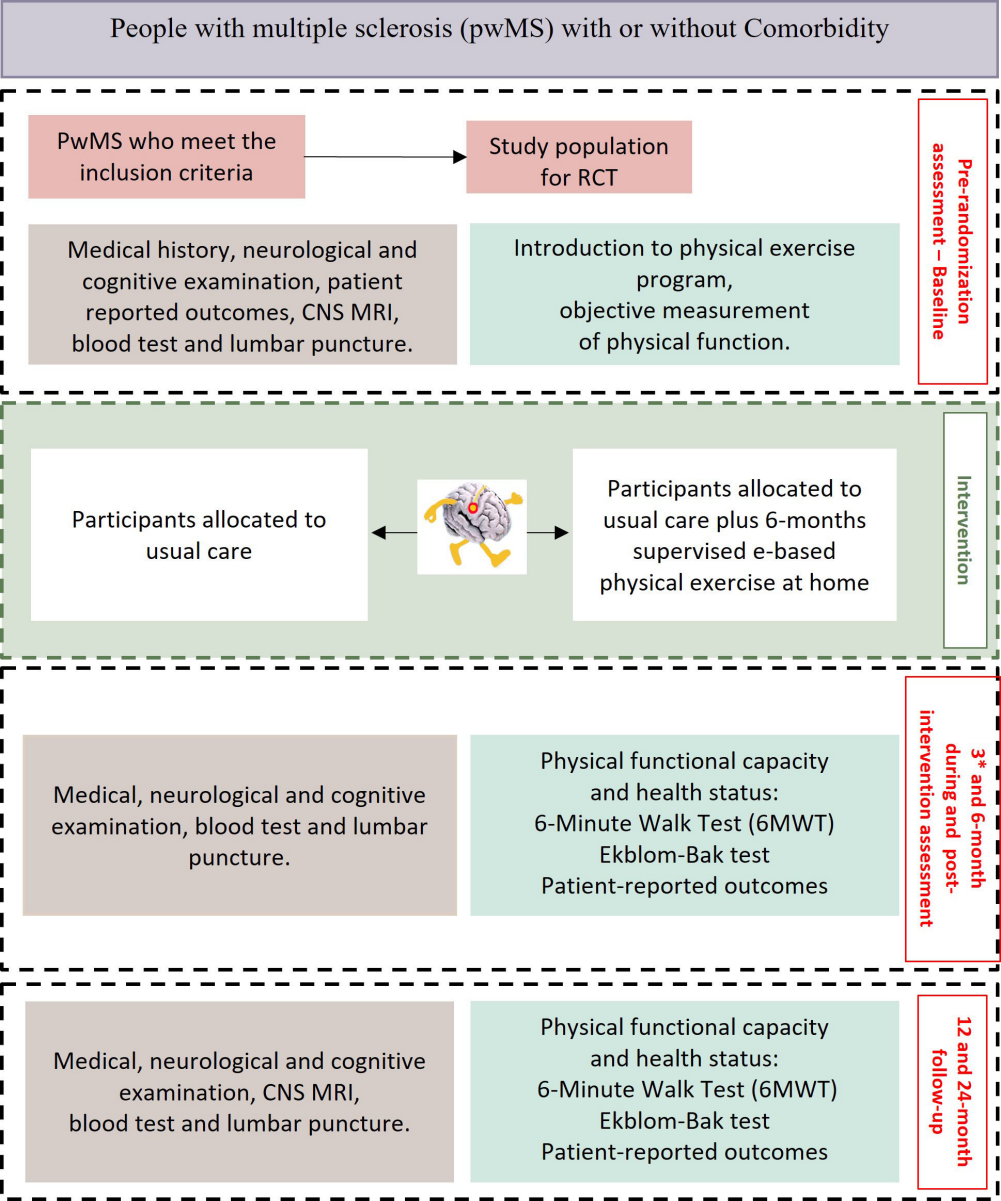
Flowchart of the randomized controlled trial design for evaluating the effects of a 6-month e-based supervised physical exercise intervention compared to usual care of patients with multiple sclerosis and comorbid conditions. The process outlines the stages from enrollment and baseline assessment until the 24-month follow-up. (*Month 3 assessments are limited to physical performance and self-reported measures. Neurological examinations, cognitive assessments, biospecimen collection, and MRI imaging are not conducted at this timepoint due to protocol constraints and participant burden considerations.).

The schematic overview of the COMPACT workflow is illustrated in **Figure 1**. This protocol delineates a RCT with prospective longitudinal data collection in pwMS with or without comorbidity. To enhance the feasibility of the main study, a feasibility study was conducted, and we report the results in this article. The study is designed according to the SPIRIT statement for the RCT and the CONSORT pilot and feasibility extension for the feasibility study (67).

### 2.2 Participants

The study will include pwMS with comorbidity (n=150) (one or more comorbidities e.g. multimorbidity) or without comorbidity (n=150). The study population will be established based on the following inclusion criteria: 1) confirmed MS diagnosis in accordance with the international McDonald Criteria for MS 2017. [3], 2) Expanded Disability Status Scale (EDSS) assessment score of less than 7.0 at baseline (68), 3) adults aged 18 years or above.

Individuals will be excluded if they have 1) current drug or alcohol abuse, 2) steroid treatment in the past month, 3) pregnancy, 4) recent trauma, active systemic infections, cancer, other chronic conditions that preclude physical exercise e.g. Parkinson’s disease or have no access/ability to use e-based solutions.

### 2.3 Recruitment procedure

Patients will be recruited from the Departments of Neurology, MS-Clinics in the Region Zealand, Denmark in collaboration with the Innovation and Research Centre for Multimorbidity at Slagelse Hospital and Department of Physiotherapy and Occupational Therapy, Slagelse. Other MS-clinics in Denmark will be requested to refer pwMS with or without comorbidity during the 2-year time period from September 1st, 2025, until May 31st, 2027. An introductory letter will be sent to the clinics, followed by further information.

### 2.4 Comorbid conditions

At the study onset comorbidity data, including diagnosis dates, characteristics, and treatments, will be extracted from patients’ medical records, and supplemented by patient interviews as needed (69, 70). In addition, each comorbidity is categorized using the International Statistical Classification of Diseases and Related Health Problem (ICD-10) System (71). For analytical purposes, comorbidity will be characterized in several ways: (1) presence versus absence of any comorbidity, (2) number of comorbid conditions (none, single comorbidity or multimorbidity defined as ≥2 comorbid conditions), and (3) type of comorbidity. Where sample size allows, exploratory subgroup analyses will be performed according to the number and category of comorbid conditions. Patients will be grouped based on the presence and type of comorbid conditions which will be clustered into six categories being cardiovascular disorders and related RF, autoimmune diseases, mental and behavioral disorders, diseases of the respiratory system, diseases of the skin and diseases of the musculoskeletal system.

### 2.5 Medical history and clinical examination

Clinical data including age, sex, ethnicity, height, weight, and lifestyle factors (e.g., tobacco and alcohol use, drug abuse history) will be recorded. Disease characteristics such as onset, current symptoms, relapse history, comorbidities, and treatments will be determined through clinical examination, interviews, and review of medical records. Disability progression will be monitored through changes in neurological status, which will be assessed with the EDSS (68), following Neurostatus definitions (72), and visual system evaluation will be conducted via best-corrected high-contrast visual acuity testing (73). Details about the initiation and cessation of disease-modifying therapies (DMT) will be recorded.

### 2.6 Blinding and randomization procedure

Patients will be randomized in a 1:1 ratio into groups receiving either usual care or usual care plus a 6-month e-based PE program, using random blocks of 4 or 6 and stratified into two groups with comorbidity and without comorbidity. Randomisation will be made blindly based on ID assigned to each patient and be stratified by comorbidity status (presence versus absence of ≥1 comorbid condition) to ensure balanced allocation across study groups. Furthermore, the patients will be stratified by age, sex, and degree of disability (EDSS) in REDCap (74). Due to the nature of the intervention, it is not feasible to blind participants or physiotherapists delivering the program. Nonetheless, to preserve the integrity of the trial, outcome assessors (researchers) will remain blinded to the allocation of treatments as well as the interpretation of the data throughout the study **Table 1**.

**Table 1:** Detailed overview of e-based exercise therapy intervention components.

Item No.	Item category	Description
1	WHAT: Materials	E-Based exercise therapy materials: elastic bands, chair, mat/blanket, camera-equipped device (computer/tablet), 65” widescreen with computer.
2	WHO: Provider	A licensed physiotherapist delivers the program.
3	HOW: Delivery	Exercises delivered in virtual groups (6–8 participants) using a wide screen and handouts.
4	HOW: Supervised/Unsupervised	Exercises are virtually supervised.
5	HOW: Measure Adherence	Attendance recorded by the physiotherapist post each session; exercise progression logged in patient diaries.
6	HOW: Motivation Strategies	Potential strategies include SMS or phone call reminders.
7	HOW: Rules for Progression	Progression guided by by Dalgas et al. (78) Details on colour of resistance band and, repetitions.
8	HOW: Replicate	Program illustrated with photographs, described in detail for each exercise level.
9	HOW: Home Program	Participants use any camera-equipped device for virtual group room access. (Tablet loans available from the hospital). Sessions hosted via Hospital-to-Citizen videoconference software (Region Zealand).
10	HOW: Nonexercise	None.
11	HOW: Adverse Events	Monitored via a daily patient diary and medical record reviews at 6-month follow-up.
12	WHERE: Location	Home-based.
13	WHEN, HOW MUCH: Dosage	60 minutes of resistance training with resistance bands twice weekly for 6 months. Details on sets, repetitions, duration, intensity. Progression model follows Dalgas et al. (78).
14	TAILORING: WHAT, HOW	Generic program with varying difficulty levels. Exercise diaries to record attendance, difficulty, sets, repetitions (78).
15	TAILORING: Decision Rule	Progression guided by established tools like OMNI scale (121)
16	Compliance: HOW WELL: Planned, Actual	The research group will test the intervention. The physiotherapist attends in another tele-rehabilitation session in order to deliver and supervise the PE program

The content is organized according to the Consensus on Exercise Reporting Template (CERT) (80) and the Template for Intervention Description and Replication (TIDieR) checklists (81), to ensure comprehensive, transparent, and reproducible reporting of the exercise and self-management intervention components.

### 2.7 Interventions

Participants in the intervention group will initially in-person receive both verbal and written instructions on using the online platform and have a one-on-one virtual session with the physiotherapist to test the setup. Resistance bands will be provided post-randomization, and tablets will be available for loan if needed. The sessions will occur on a secure platform used by Region Zealand, tested and proven feasible in a mixed-methods study in patients with osteoarthritis and comorbidity (75).

**Figure 2:**
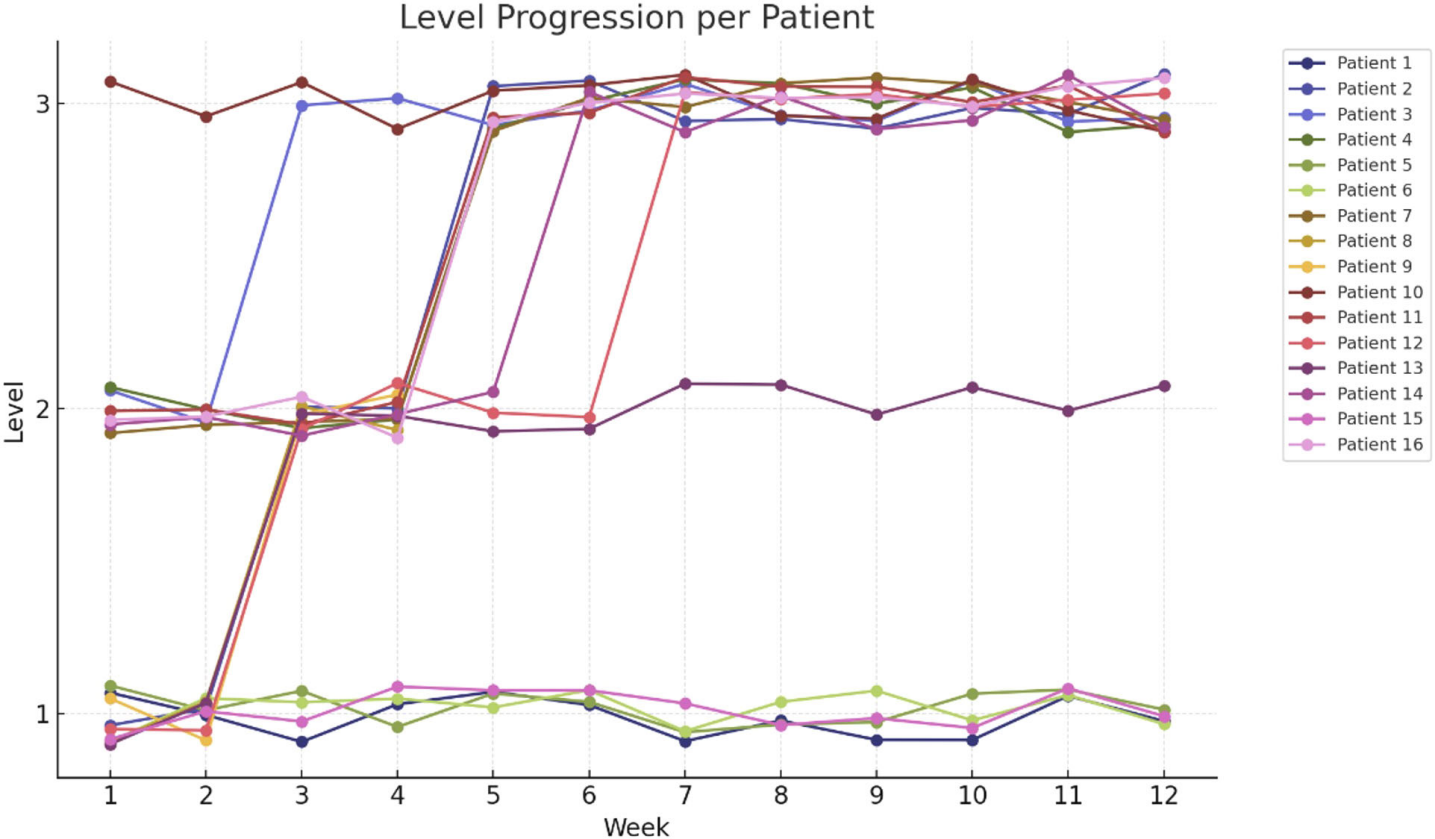
Level Progression per Patient. Each line (with circular markers) represents one of the 16 patients’ weekly progression through exercise levels 1–3 over a 12-week protocol. Vertical jitter has been applied to markers to prevent overlap, and each patient is assigned a distinct color. The x-axis shows Week 1–12; the y-axis shows Level (1 = easy, 3 = hard).

**Figure 3:**
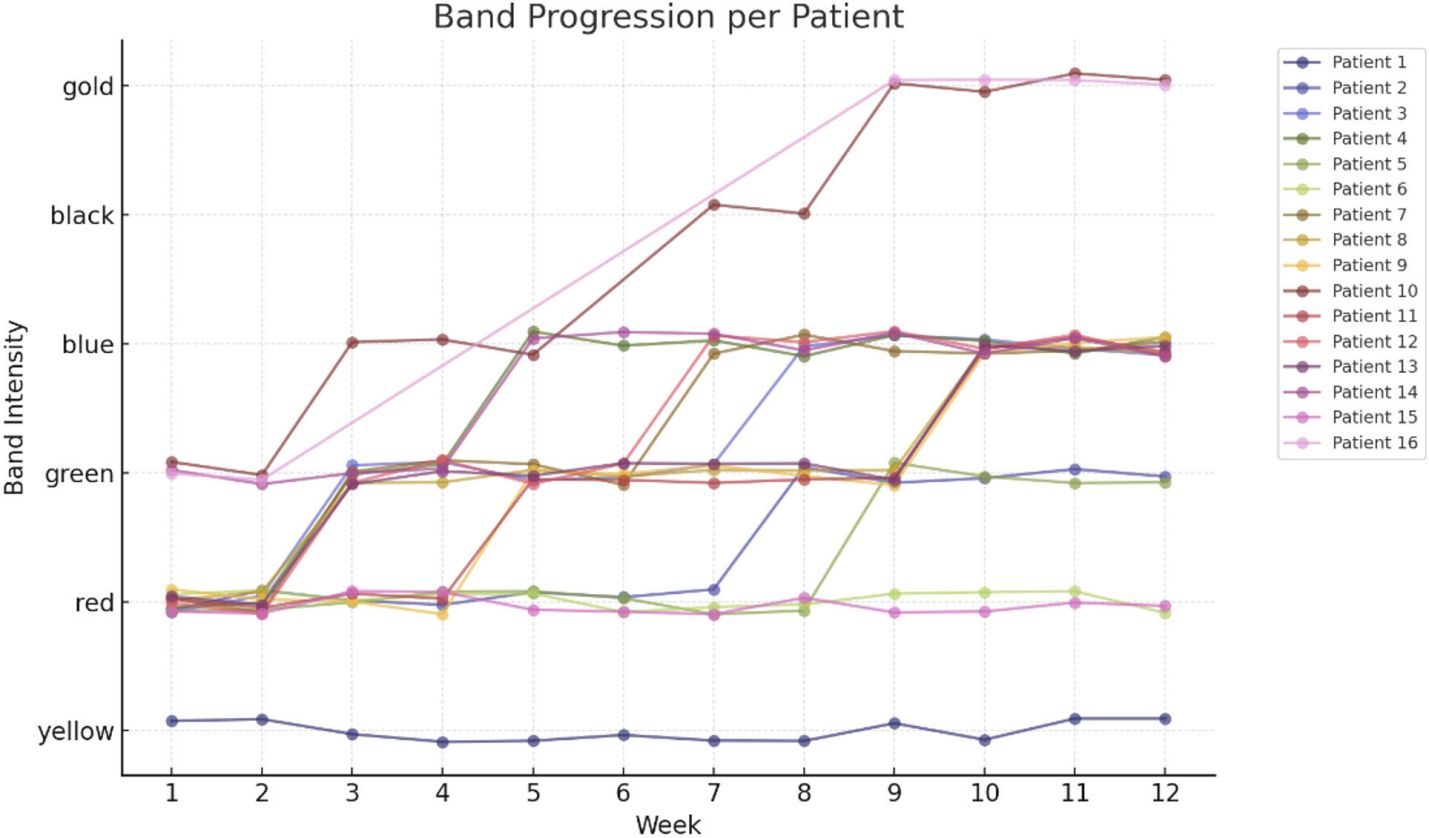
Band Progression per Patient. Each line (with circular markers) tracks one patient’s progression through theraband resistance bands (yellow→red→green→blue→black→gold) over the same 12 weeks. Vertical jitter separates overlapping points; colors match those in **Figure 1** for patient consistency. The x-axis shows Week 1–12, and the y-axis is labeled with band names in order of increasing resistance.

**Figure 4:**
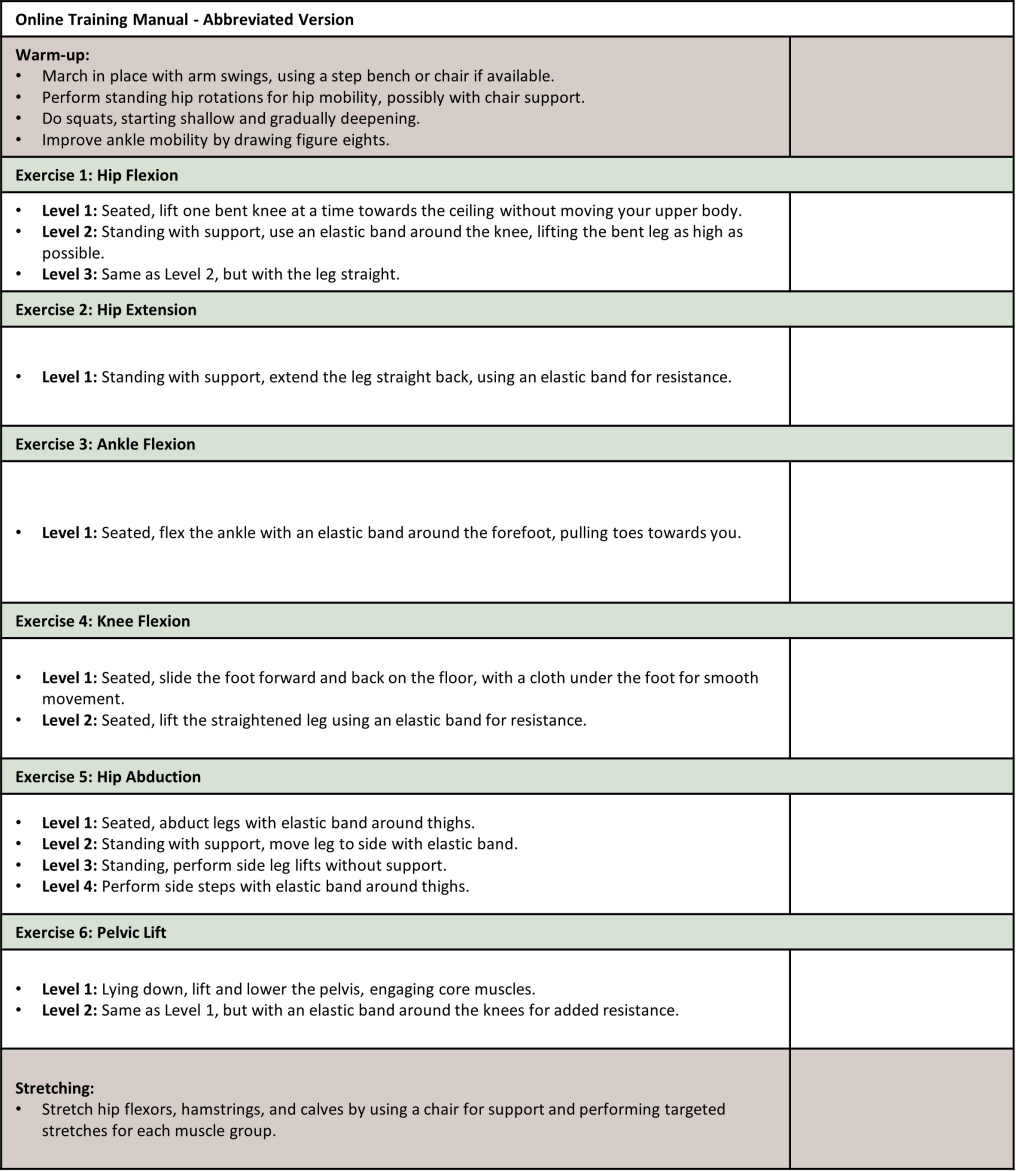
A comprehensive guide for a full-body warm-up and exercise routine aimed at enhancing joint mobility and muscle flexibility. The routine is segmented into six core exercises, each targeting specific areas of the body, supplemented by a warm-up sequence and a stretching sequence for overall muscle preparedness and recovery.

The e-based exercise intervention is comprised of 6-months of supervised home-based resistance training for the lower extremities, conducted twice weekly **Figure 2**. The focus on the lower extremities is due to the pronounced impairment of lower extremity muscle strength compared to the upper extremities in pwMS (76). The intervention builds on two previous studies on supervised progressive resistance training for pwMS, which effectively improved walking performance (77, 78). The original protocol is modified to e-delivery (78), incorporating six exercises including, hip flexion, hip abduction, knee flexion and extension, ankle dorsal flexion, and gluteal bridge, using resistance bands **Figure 3**. Furthermore, resistance band training has been beneficial for the upper extremity in pwMS in home-based training programs (79) (**Figure 4**).

All 48 sessions will be conducted in virtual groups of 6–8 participants, supervised by an experienced physiotherapist, connected to the virtual group from an exercise facility at the hospital using a 65” widescreen with computer hardware. Participants will perform exercises with a fast concentric phase and a slow eccentric phase. The load will be adjusted using different resistance bands. The progression model for sets, repetitions, and load follows the approach used by Dalgas et al. (78), which specifies: week 1–4 with 3 sets of 10–12 repetitions at a load of 15 repetitions maximum (RM), week 5–8 with 3 sets of 12 repetitions at a load of 12 RM, week 9–12 with 4 sets of 12 repetitions at a load of 12 RM, week 13–16 with 4 sets of 10 repetitions at a load of 10 RM, week 17–20 with 4 sets of 8 repetitions at a load of 8 RM, and week 21–24 with 3 sets of 8 repetitions at a load of 8RM. The participant is allowed 2 to 3 minutes of rest between sets and exercises.

Usual care refers to routine MS physiotherapy provided once weekly in accordance with Danish clinical practice. All care received during the trial period, including any additional physiotherapy sessions, will be systematically recorded to ensure transparency and comparability across groups.

The details of the exercise therapy component, including activities, duration, frequency, and methodologies, are reported in accordance with the Consensus on Exercise Reporting Template (CERT) (80), ensuring clarity and replicability. For the self-management component, the intervention is described following the Template for Intervention Description and Replication (TIDieR) checklist (81). A comprehensive overview of both components is provided in 1.

### 2.8 Adherence measurement and motivation strategies

Attendance is recorded by the physiotherapist after each session. Additionally, patients are required to log their exercise progression in personal diaries, ensuring comprehensive tracking of their engagement and progress. We will employ strategic communication methods to bolster participation and adherence, including SMS reminders and phone calls. These reminders serve not only to motivate but also to provide timely prompts for upcoming sessions, thereby aiming to enhance overall engagement and consistency in participation.

Adherence to the intervention will be assessed at the 6-month follow-up as the number of exercise sessions attended out of the total number of sessions available. Satisfactory adherence will be attained when completing 36 out of 48 sessions (75%).

### 2.9 Outcome parameters and assessment timeline

Outcome measures and assessment timeline are depicted in **Table 2**. In brief:

**Table 2:** Timeline of key assessments and measurements throughout the study period.

Assessment	Baseline	3 M (PE)	6 M (PE)	12 M	24 M
Physical excerise randomised clinical Trial					
**Gait Impairment:** 6-Minute Walk Test (6MWT), MS Walking Scale 12 (MSWS-12)	✓	✓	✓	✓	✓
**Measurement of Physical Activity:** International Physical Activity Questionnaire-Short Form (IPAQ-SF)	✓	✓	✓	✓	✓
**Cardiorespiratory Fitness:** Ekblom-Bak test (sub-maximal cycle ergometer test)	✓	✓	✓		
**Adverse Events (AE) and Serious Adverse Events (SAE):**	✓	✓	✓	✓	✓
**Adherence to Intervention:** the proportion of attended sessions to total sessions available	✓	✓	✓	✓	✓
Prospective longitudinal data collection					
**Demographics:** Age (year), Sex, Race/Ethnicity, Geographic location, Height (cm), Education level, Marital status, Work status	✓				
Weight (Body Mass Index (BMI), Waist Circumference)	✓	✓	✓	✓	✓
**Medical history:** Diagnosis, Disease onset, Attack/Relapse History (Number of Relapse(s)**), Current Symptoms and Complaints, Vaccination History, Previous Infections; Fertility History, Previous injuries/surgeries, Family medical history, Dietary and Lifestyle Factors, Treatment’s status; Relapse Therapy; Full Drug List	✓	✓	✓	✓	✓
**Comorbid conditions:** Date of diagnosis, the severity (when available), the number of comorbid conditions grouped by main categories, and the actual raw numbers.	✓	✓	✓	✓	✓
**Risk factors:** Smoking, Alcohol consumption,	✓	✓	✓	✓	✓
**Neurological Examinations:** EDSS***, Hand grip force (hand−held dynamometer, 3x each side), Timed tests (T25−FW, 9−HPT), Visuo−perceptive motion analysis PASS−MS (short walks, static balance, stand−up and sit, stepping in place, finger tapping, finger−nose test) Spasticity assessment (Modified Ashworth Scale)	✓		✓	✓	✓
**Patient Reported Outcomes (PRO):** EuroQol 5-Dimension 5-Level Scale (EQ-5D-5L); Fatigue Severity Scale (FSS); International Physical Activity Questionnaire–Short Form (IPAQ-SF); Insomnia Severity Index (ISI); Modified Fatigue Impact Scale (MFIS); Patient Health Questionnaire-8 (PHQ-8); Pittsburgh Sleep Quality Index (PSQI); Visual Analog Scale (VAS).	✓	✓	✓	✓	✓
**Cognitive assessment:** Interview, Montreal Cognitive Assessment (MoCA), Symbol Digit Modalities Test (SDMT)	✓		✓		✓
Biospecimens* Blood samples, Cerebrospinal Fluid	✓		✓	✓	✓
CNS magnetic-resonance imaging^*^ Cerebral: MPRAGE, T2−SPACE, FLAIR, MPM, DWI, rsfMRI Spinal: STIR (whole spine), PSIR (C2&C3 and C7/T1)	✓				✓

*Further details can be found in the method section.

** Number of Relapse(s) (a new or worsening acute neurologic symptom lasting ≥24 hours and not explained by fever or infection).

*** Disability assessment with alteration in EDSS score of at least 0.5 from baseline at the clinical visits.

"no evidence of disease activity" (NEDA-3) score will be calculated using data collected on relapses, EDSS, and MRI findings from the study.

1. The 6-Minute Walk Test (6MWT): The 6-Minute Walk Test (6MWT), as the primary outcome, quantitatively measures walking capacity, reflecting an individual’s endurance and mobility by recording the distance covered within six minutes on a hard, flat surface (82, 83).
2. Physical exercise and cardiorespiratory fitness: The assessment framework encompasses a multifaceted approach to evaluating PE and cardiorespiratory fitness. The Ekblom-Bak test, a sub-maximal cycle ergometer assessment, will be employed to estimate maximal oxygen consumption (VO2 max). This test indirectly measures an individual’s cardiorespiratory fitness by analyzing the heart rate response to incremental exercise, which is a critical determinant of aerobic fitness and overall cardiovascular health (84, 85).
3. Physical activity monitoring: Objective measurement of physical activity (PA) by accelerometers will be performed at baseline and at finalization of the study. Two Axivity AX3 accelerometers will be used to measure PA in each participant, who will wear the accelerometers attached to the skin, one attached to the front of the thigh using medical tape and the other to the non-dominant wrist using wristbands. In brief, skin-taped Axivity AX3 accelerometers have shown high compliance based on 24-h wear-time and has high data quality (86).
4. Spasticity assessment: Spasticity will be evaluated using the Modified Ashworth Scale at baseline and at 6, 12, and 24 months. This standardized measure will allow for quantification of lower-extremity muscle tone and stiffness, complementing gait and disability assessments.
5. Disability assessment: Disability assessment using EDSS with alteration in score of at least 0.5 from baseline regarded as significant.
6. Relapse rate: Number of Relapse(s) (a new or worsening acute neurologic symptom lasting ≥24 hours and not explained by fever or infection).
7. MRI of CNS: assessment of new or enlarged T2W or enhancing lesion indicating radiological diseases activity.
8. Cognitive function: Cognitive functions will be evaluated using the Montreal Cognitive Assessment (MoCA). Participants’ education level and hand preference will be assessed through interviews (87).
9. Patient-reported outcomes (PROMs): PROMs will be measured as a) PA level will be evaluated using the International Physical Activity Questionnaire-Short Form (IPAQ-SF) to categorize 3 groups of low, moderate, or high level of PA (88), b) current level of fatigue by the Modified Fatigue Impact Scale (MFIS) (89) and the Fatigue Severity Scale (FSS), c) quality of life using the European Quality of Life–5 Dimensions (EQ-5D-5L) (90) and d) depression level will be measured using the Personal Health Questionnaire Depression Scale (PHQ-8) (91) e) sleep quality and disturbances will be measured using the Pittsburgh Sleep Quality Index (PSQI) and the Insomnia Severity Index (ISI), which assess overall sleep quality and the severity of insomnia (92), respectively. f) Symptom intensity will be captured using a Visual Analog Scale (VAS), allowing participants to rate their perceived severity of pain.

### 2.10 Biospecimens

Blood and CSF samples will be systematically collected at four key time points: baseline (prior to starting the exercise program), at 6 months (upon completion of the program), at 12 and 24 months. All clinical and laboratory data derived from these samples will be analyzed while maintaining blinding to ensure unbiased assessment. The markers of inflammation, metabolism and neurodegeneration include matrix metallopeptidase (MMP), vascular endothelial growth factor A (VEGF-A) and microfibril-associated protein 4, measured by ELISA. Selected cytokines interleukin (IL)-1beta, IL-6, IL-7, IL-10, IL-17A, tumor necrosis factor alpha (TNF-alpha), and NfL, Chitinase-3-Like-1, and Type I interferons, will be measured on a Quanterix™ Simoa HD-1 Analyzer using appropriate Simoa assay kits (93).

### 2.11 MRI

Participants will undergo initial and final CNS MRI using a 3T system. This includes brain, spinal cord, and optic nerve imaging with sequences like 3D FLAIR, T2 SPACE, and T1 MPRAGE. Advanced techniques like multi-parametric mapping and diffusion-weighted imaging will be employed for detailed CNS characterization, focusing on changes related to MS and vascular comorbidities (94, 95).

### 2.12 Adverse events

Adverse events (AE) will be systematically documented for both the intervention and the usual care group with a daily, pre-printed patient diary over the course of the 6-month period. Patients will report any symptoms or pain experienced post-PE. Both AE and serious adverse events (SAE) will be registered at all follow-up visits, employing open-ended questions to ensure comprehensive recording of all AEs. Additionally, patient medical records will be reviewed at 3 months during the program and at the primary endpoint (6 months) to identify any AEs that have occurred since enrollment. AEs will be classified in accordance with the Food and Drug Administration’s definition of SAE. The recording, categorization, and assessment of the severity of AEs will be conducted irrespective of presumed causality with study treatments. These evaluations will be conducted at 6 months post-intervention.

### 2.13 Data management

The data related to this study will be stored in the REDCap database system (96). This electronic platform is chosen for data collection and storage to ensure optimal data uptake.

### 2.14 Data monitoring and auditing

Monthly data monitoring will be conducted using a REDCap query function for completeness and accuracy by the research team to ensure that any errors or inconsistencies are identified and addressed in a timely manner.

### 2.15 Outcome measures

The primary endpoint will be evaluation of walking capacity by 6MWT from baseline to end of the PE program: (at baseline, 3, 6, 12 and 24 months).

Secondary outcomes include a “no evidence of disease activity” (NEDA-3) score characterized by three parameters 1) lack of clinical relapses, 2) disease progression measured by EDSS, and 3) MRI lesion data, e.g. absence of new T2 lesion/enhancing lesion, after two years’ observation (97–100). In addition, secondary endpoints include measures of quality of life and fatigue, as well as levels of cardiorespiratory fitness and neurofilament light chain (NfL) levels.

The role of comorbidity within the study design, including use as a stratification variable during randomization and as a prespecified covariate and potential effect modifier in the statistical analyses. In addition, the Charlson Comorbidity Index (CCI) will be used to quantify comorbidity burden in pwMS (101).

The efficacy of the interventions on comorbidities will be determined by using a combination of both objective physical tests, patient-reported outcomes and clinical/biochemical markers from baseline to post-intervention to assess outcome.

### 2.16 Ethical considerations

The study was approved by The Regional Committee on Biomedical Research Ethics (Ref. no. S-20230011) and registered by ClinicalTrials.gov registration: NCT06298201. In addition, the study will be registered at the Danish Data protection agency. It will be conducted in accordance with the guidelines for good clinical practice set forth by the International Conference on Harmonization.

### 2.17 Sample size calculation

A sample size calculation for the PE RCT was conducted to assess the effect of the intervention on the primary outcome, the 6-Minute Walk Test (6MWT), while accounting for comorbidity status and any potential interaction between comorbidity and the intervention. The calculation was based on:

1. A two-way factorial design incorporating intervention group (PE vs. control) and comorbidity status (yes/no) as binary factors, with a binary outcome based on the 6MWT, reflecting whether participants achieved a clinically meaningful improvement.
2. A logistic regression model including intervention, comorbidity, and their interaction term as covariates.

The null hypothesis (H_0_) of no treatment effect was tested against the alternative hypothesis (H_1_) as either a main effect of the intervention or a significant intervention–comorbidity interaction, using a chi-square test derived from the logistic regression model.

Assumptions for the calculation were as follows: in the control group, it was assumed that 40% of participants with comorbidity and 20% without comorbidity would fail to achieve a clinically meaningful improvement in 6MWT. In contrast, in the intervention group, these proportions were expected to be reduced to 10% and 20%, respectively. This corresponds to a relative risk of 0.5, which is considered clinically significant.

Based on 10,000 simulation runs, a total of 73 participants per combination of intervention and comorbidity status (i.e., 292 participants in total) would be required to achieve approximately 80% statistical power at a 5% significance level. The simulations were performed using R software, version 4.1.2.

### 2.18 Statistical analysis

All data generated and collected during the project will undergo comprehensive statistical analysis using both traditional and advanced modeling techniques. The analyses will follow the intention-to-treat (ITT) principle as the primary analytic strategy. A per-protocol (PP) analysis will be conducted as a secondary approach, restricted to participants in the intervention group who meet the following criteria: (1) attendance of at least 75% of the prescribed exercise sessions, (2) completion of at least one follow-up assessment (e.g., at 6 months), and (3) submission of required self-report data such as exercise diaries and PROMs.

To evaluate the efficacy of the PE intervention, longitudinal changes in outcomes such as exercise capacity, functional abilities, and patient-reported measures will be analyzed using a multivariable regression model.

These models account for repeated measurements within individuals over time and are well suited to the handling of missing data under a missing-at-random assumption.

In parallel, changes in clinical disease biomarkers and standard clinical outcomes (e.g. EDSS, relapse rate) will also be examined using mixed-effects models for both within-group and between-group comparisons. Particular attention will be given to biomarkers that reflect MS activity or progression (e.g., serum GFAP, NfL), with adjustment for relevant covariates such as age, sex, and disease duration.

Key statistical analyses will include:

1. Association analyses, where logistic regression models will be used to assess the relationships between clinical outcomes and explanatory variables such as comorbidity status, age, and sex, including testing for potential interaction effects.
2. Correlation analyses, where Pearson or Spearman correlation coefficients will be estimated to explore associations between multiple clinical endpoints, aiding in the interpretation of the multidimensional impact of MS.
3. Predictive modeling of disease progression, which will be pursued as part of a follow-up analysis. We aim to construct models to predict MS progression by integrating disease biomarkers and vascular comorbidity risk factors. To this end, lasso logistic regression models will be employed, and model performance will be assessed using Area Under the Curve (AUC) metrics.

During the current study phase, preliminary steps will focus on feature selection and initial model training, which will lay the groundwork for more comprehensive models in future phases. Collectively, these statistical approaches are intended to provide a robust, multidimensional understanding of MS progression and the role of PE interventions and comorbidities in shaping long-term outcomes.

In the feasibility study for the progression model for PE level and band intensity a linear mixed-effects model was fitted separately for PE level (1–3) and for band code (1 = yellow…6 = gold), each with week (1–12) as the sole fixed effect and with random intercepts and random slopes by patient to account for individual baselines and progression rates.

### 2.19 Feasibility study

Before the main RCT, a feasibility study was performed to increase the feasibility of the main study. This randomized controlled feasibility trial included pwMS, with and without comorbidity, who were followed from June 1^st^, 2024, to April 1^st^, 2025, in a real-world setting and followed the same procedures as described above for the RCT. Participants were randomly allocated in a 1:1 ratio to either a digital PE intervention group or a usual care control group using block randomization with variable block. Fifty individuals were eligible and randomized into an intervention group (n = 23) and a usual care control group (n = 27) (**Table 3**).

**Table 3:** Baseline characteristics of participants in the feasibility study.

Characteristic:	Total (n = 50)	Intervention (n = 21/23)	Usual care (n = 27)	P-value†
Age, years (mean ± SD)	58.7 ± 11.3	58.7 ± 11.3	57.1 ± 12.0	0.45 ‡
Female sex, n (%)	32 (64%)	15 (71.4%)	17 (63%)	0.54 §
EDSS, median	3.5	3.5	3.5	0.91 ¶
MS phenotype, n (%)				
– Relapsing–remitting MS (RRMS)	24 (48%)	11 (52.4%)	13 (48.1%)	0.77 §
–Progressive MS (Primary or Secondary)	24 (48%)	10 (47.6%)	14 (52%)	
Presence of ≥1 comorbidity, n (%)	26 (52%)	9 (43%)	17 (63%)	0.17 §

^†^p-values refer to comparisons between intervention and usual-care groups.

^‡^Independent-samples t-test.

^§^Chi-square test or Fisher’s exact test (as appropriate).

^¶^Mann–Whitney U test.

### 2.20 Results of the feasibility study

Participants in the intervention group at baseline received education on using the online platform and had an individual virtual session with the physiotherapist to test the setup. All 24 sessions were conducted within three months in virtual groups of 6 participants, supervised by a trained physiotherapist. Continuous communication and supervision were provided by the physiotherapist.

In the digital PE group, 23 participants were enrolled, two did not begin the PE program due to occupational constraints, yielding a recruitment rate of 91.3% (21/23). Of these, 16/21 (76.2%) completed the follow-up assessments, five participants discontinued participation due to competing personal demands including spousal illness and familial health burdens (n=3) as well as participant-specific factors such as auditory deficits (n=1), and occupational constraints (n=1). No intervention‐related adverse events (AEs) were reported.

Adherence rate and compliance: the 16 participants who completed follow-up collectively attended 243 sessions. This results in a mean attendance of 15.2 (range 6-22) sessions per participant out of the planned 24 sessions, corresponding to a 63.33% adherence rate.

Baseline characteristics of the intervention group (n = 21/23) showed a mean age of 58.7 years (SD 11.3), 42.8% of participants had ≥1 comorbidity, and the median EDSS was 3.5 (IQR 3.75).

#### 2.20.1 The progression model for PE level and band Intensity in the feasibility study

Progression in PE Level and Band Intensity were analyzed for the pwMS in the feasibility study, using two statistical models (**Table 4**): 1) Level model: The intercept was estimated as 1.55 (SE = 0.15; 95% CI 1.25–1.85), and the effect of week as β = 0.09 (SE = 0.02; 95% CI 0.05–0.13; z = 4.42; p < 0.001), indicating an average increase of 0.09 level-units per week.

**Table 4:** Mixed-effects model estimates for weekly progression in exercise level and band intensity in the feasibility study.

Model	Term	Est.	SE	z	p	95% CI
Level	Intercept	1.55	0.15	10.11	5.22 × 10^-24^	[1.25, 1.85]
	Week	0.09	0.02	4.42	9.80 × 10^-6^	[0.05, 0.13]
Band	Intercept	2.01	0.16	12.76	2.64 × 10^-37^	[1.70, 2.32]
	Week	0.15	0.04	3.66	2.49 × 10^-4^	[0.07, 0.24]

This table presents results from mixed-effects models assessing changes in exercise level and resistance band intensity over a 12-week training protocol. Both models show statistically significant upward trends, indicating consistent weekly increases. The estimates include fixed-effect intercepts and weekly slope coefficients (β), along with standard errors (SE), z-values, p-values, and 95% confidence intervals (CI). The models account for repeated measures within individuals over time.

2) Band-code model: The intercept was estimated as 2.01 (SE = 0.16; 95% CI 1.70–2.32), and the effect of week as β = 0.15 (SE = 0.04; 95% CI 0.07–0.24; z = 3.66; p < 0.001), indicating an average increase of 0.15 band-code units per week.

Mixed‐effects progression modeling determined a statistically significant upward trajectory for both exercise level (β = 0.09 ± 0.02 units/week, p < 0.001) and band intensity (β = 0.15 ± 0.04 units/week, p < 0.001) over the 12−week protocol (**Table 4**).

#### 2.20.2 Patients’ experiences with the feasibility study

By an individual conversation after exercise at the feasibility study the participants expressed their thoughts. They emphasized that the daily patient diary documentation for monitoring AE needed professional supervision. Participants experienced challenges incorporating digital exercise into their work-life routines. Observations further suggested that groups with having comparable age and PA levels tended to perform more cohesively. The positive experiences included the ease of logging in, the value of communication and the supervision performed by the physiotherapist. Moreover, a short structured post-exercise discussion with physiotherapist appeared to enhance social interaction and motivation.

## 3 Discussion

This protocol describes a prospective RCT study designed with longitudinal follow-up of pwMS with and without comorbidities, utilizing a standardized comorbidity-focused algorithm to explore the efficacy of e-based PE interventions in addition to usual care on clinical and paraclinical outcomes. The methodology includes standardized data collection on comorbid conditions such as recording the date of diagnosis, verifying diagnoses, assessing disease duration, a clinical examination and assessment of comorbidities, determination of functional capacity by 6MWT, and evaluation of cardiorespiratory fitness by the Ekblom-Bak test, a sub-maximal cycle ergometer assessment, estimating the maximal oxygen uptake (VO2-max). Finally, pwMS will complete a range of patient-reported health status questionnaires. at baseline and end of study. We have tested this structured approach in a feasibility study to enhance the rigor of the main RCT and results support to proceed to RCT. Fifty pwMS were eligible and randomized into the usual care control group or to the intervention group with an overall recruitment rate of 91.3%. In the intervention group 76.2% completed the follow-up assessments and had complete outcome data. Determination of functional capacity and evaluation of cardiorespiratory fitness were performed at baseline and at the end of the study. No intervention‐related SAEs were reported. Tracked adherence using a diary or logbook as the participants logged into the computer for the exercise session showed a 63.3% adherence rate. Attendance and progress were rigorously monitored, with physiotherapists recording each session’s attendance and participants logging their exercise progression into personal diaries. This dual tracking system ensured a detailed overview of engagement and advancements. Moreover, we used strategic communication techniques to boost participation and adherence. Using SMS reminders and phone calls, we motivated participants and provided them with timely notifications about upcoming sessions, significantly enhancing engagement and consistency in participation. Despite this thorough approach, the feasibility study had a 23.8% (5/21) drop-out, the most common reasons being lack of time and support due to illness in the family and occupational constraints. These challenges underscore the need for enhanced flexibility of scheduling and tailored support such as recording of the PE programme for later performance. In the feasibility study the progression models showed statistically significant upward trends, indicating consistent weekly increases. The progression models may improve functional capacity in assessment in the future RCT study.

Current management of MS is non-curative. The immunotherapies currently approved for MS act on the inflammatory component of the disease (Disease-Modifying Therapies (DMT)) and reduce clinical/radiological relapses in relapsing-remitting course (RRMS). While these DMTs effectively mitigate clinical and radiological relapses, they are largely insufficient to prevent disease progression or the accumulation of disability (5). RRMS frequently transforms into a secondary progressive course, marked by steady neurologic decline and disability due to axoneuronal damage (4). Moreover, the increasing age-specific prevalence of MS over recent years carries an augmented risk of comorbidities and advancement to more debilitating disease stages, underscoring the need for comprehensive treatment approaches that address both the inflammatory and neurodegenerative components of MS (2, 6, 7). PE has emerged as a potentially effective therapeutic strategy for pwMS (13, 18, 61, 102, 103). The success of PE in managing MS symptoms and impeding disease progression is highly dependent on the specific characteristics of the exercise intervention, such as the type, intensity (104), frequency and duration of exercises (105). This underscores the essential need of exercise programs to be carefully tailored to the unique requirements and disease profiles of each patient. Despite the increasing knowledge that PE has benefits for pwMS, high drop-out rates from PE, low maintenance of recommended levels of PE at follow-up and inactivity have been observed (53, 106), suggesting barriers to integrating PE in the daily life of pwMS. Identifications of the variables associated with engagement in PE may facilitate the development of interventions focusing on adherence. One of the primary challenges in implementing PE interventions lies in the considerable variability among patients. MS manifests with a wide range of symptoms and degrees of disability, necessitating highly individualized exercise plans (105). Fatigue, a prevalent symptom among pwMS, can significantly hinder engagement and adherence to these exercise regimens (107, 108). Mobility issues further complicate the scenario, often requiring specialized equipment or modifications to ensure safe and effective participation (61, 109). Accessibility to specialized facilities and trained professionals also presents a significant barrier, limiting the availability of tailored exercise opportunities for many, and may explain part of the frequent drop-out (110, 111). There is heterogeneity in the outcome of rehabilitation interventions in pwMS and the presence of comorbidity may explain this observation, complicating PE rehabilitation programs and reducing efficacy of the trials (63, 112).

In response to these challenges, digital exercise interventions, including online programs and virtual reality exercises, have been developed to offer a more flexible and accessible option for pwMS (113–115). However, these digital solutions are not without challenges and limitations. their own set of obstacles. Technology access remains a hurdle, as not all patients have the necessary devices or internet connection. Although digital platforms provide an innovative approach to exercise, they often lack the level of personalization and adaptability that comes with direct interaction with healthcare professionals. Furthermore, maintaining motivation and engagement in a digital format can be daunting without the encouragement and support of an instructor (106, 116, 117).

There are also safety concerns to consider; without professional supervision, there is an increased risk of injury or performing exercises incorrectly, especially for those with significant physical limitations (118, 119). These complexities and the need for highly personalized care are particularly pronounced in cross-sectional studies, which may not fully capture the longitudinal dynamics of patient responses to exercise or the nuanced interplay of MS symptoms and exercise efficacy.

The COMPACT research program investigates e-based PE at home for pwMS and comorbidities including long-term follow-up. To address and potentially overcome many of the identified limitations, the COMPACT program incorporates several strategies (120).

Education, supervision and communication with the participants will be performed at baseline and continue with the physiotherapist. Baseline assessment of physical function level and mental status will be performed. As comorbidities, especially cardiovascular disorder and related risk factors, are common in pwMS and influence clinical outcomes, this study will create a uniform diagnostic and prognostic methodology to elucidate the effect of comorbidity on clinical and paraclinical outcomes in pwMS. The present study is expected to provide novel knowledge that will benefit clinical practice on how to implement e-based delivery of PE in pwMS, particular with comorbidities on top of MS thereby introducing a holistic rehabilitation strategy.

## Data Availability

The raw data supporting the conclusions of this article will be made available by the authors, without undue reservation.
